# Are local public expenditure reductions associated with increases in inequality in emergency hospitalisation? Time-series analysis of English local authorities from 2010 to 2017

**DOI:** 10.1136/emermed-2022-212845

**Published:** 2024-06-13

**Authors:** Ana Cristina Castro-Ávila, Richard Cookson, Tim Doran, Robert Shaw, John Brittain, Sarah Sowden

**Affiliations:** 1 Health Sciences, University of York, York, North Yorkshire, UK; 2 Carrera de Kinesiologia, Universidad del Desarrollo Facultad de Medicina Clínica Alemana, Santiago, Chile; 3 Centre for Health Economics, University of York, York, UK; 4 NHS England and NHS Improvement London, London, UK; 5 Population Health Sciences Institute, Newcastle University, Newcastle, UK

**Keywords:** admission avoidance

## Abstract

**Background:**

Reductions in local government funding implemented in 2010 due to austerity policies have been associated with worsening socioeconomic inequalities in mortality. Less is known about the relationship of these reductions with healthcare inequalities; therefore, we investigated whether areas with greater reductions in local government funding had greater increases in socioeconomic inequalities in emergency admissions.

**Methods:**

We examined inequalities between English local authority districts (LADs) using a fixed-effects linear regression to estimate the association between LAD expenditure reductions, their level of deprivation using the Index of Multiple Deprivation (IMD) and average rates of (all and avoidable) emergency admissions for the years 2010–2017. We also examined changes in inequalities in emergency admissions using the Absolute Gradient Index (AGI), which is the modelled gap between the most and least deprived neighbourhoods in an area.

**Results:**

LADs within the most deprived IMD quintile had larger pounds per capita expenditure reductions, higher rates of all and avoidable emergency admissions, and greater between-neighbourhood inequalities in admissions. However, expenditure reductions were only associated with increasing average rates of all and avoidable emergency admissions and inequalities between neighbourhoods in local authorities in England’s three least deprived IMD quintiles. For a LAD in the least deprived IMD quintile, a yearly reduction of £100 per capita in total expenditure was associated with a yearly increase of 47 (95% CI 22 to 73) avoidable admissions, 142 (95% CI 70 to 213) all-cause emergency admissions and a yearly increase in inequalities between neighbourhoods of 48 (95% CI 14 to 81) avoidable and 140 (95% CI 60 to 220) all-cause emergency admissions. In 2017, a LAD average population was ~170 000.

**Conclusion:**

Austerity policies implemented in 2010 impacted less deprived local authorities, where emergency admissions and inequalities between neighbourhoods increased, while in the most deprived areas, emergency admissions were unchanged, remaining high and persistent.

**WHAT IS ALREADY KNOWN ON THIS TOPIC**

- Local government spending reductions increase mortality and inequality in mortality.
- However, the impact of local government spending on emergency admissions is not known.
- One previous study found no association but did not account for expected increases in funding and growing levels of need or break down the findings by deprivation group to look at between or within area inequality impacts.

**WHAT THIS STUDY ADDS**

- Austerity policies implemented in 2010 had an effect on less deprived local authorities where emergency admissions and inequalities between neighbourhoods increased, while for the most deprived, they remained high and persistent.

**HOW THIS STUDY MIGHT AFFECT RESEARCH, PRACTICE OR POLICY**

- Reductions in local government funding had a detrimental effect not only on mortality but also on emergency admissions in less deprived areas.
- Government policies counteracting negative economic conditions should consider the long-term impact of worsening population health on the emergency medicine and social care systems, and the extra costs this might produce.

## Introduction

People in socioeconomically deprived communities are more likely to develop illness and less likely to receive healthcare proportionate to their needs.^1–3^ In England, this situation has been exacerbated by funding decisions taken after the 2008/2009 recession, with local authorities in the most deprived areas facing the greatest absolute reductions in funding.^4 5^

People living in more deprived places are more likely to have an emergency hospital stay and also one that could have been avoided. ‘Avoidable admissions’ are those for which timely and effective ambulatory care can prevent the need for hospitalisation.^6 7^ Alongside age, socioeconomic deprivation is the strongest risk factor for avoidable hospital admission.^8^ In 2015, the inequality gap between England’s most and least deprived areas was estimated to produce an excess of 263 894 emergency admissions.^9^

Inequalities in health and healthcare cannot be addressed by healthcare systems alone; concerted action on the wider determinants of health, with investment in strategies that keep people healthy in the first place, including housing, education, employment and social support networks, is critical.^3 10^ Much of this activity, including delivering social care, is discharged through local government. Funding reductions for these services will likely negatively impact local government delivery of activities that support health and well-being in the community.

There is considerable evidence of the adverse impact local government funding reductions are likely to have on mortality and inequalities in mortality but much less evidence about the impacts on emergency hospital admissions. Local government spending reductions during the 2010s were associated with declines in life expectancy and increases in national inequalities in life expectancy.^11^ Associations have also been found with worsening multimorbidity and health-related quality of life.^12^ Furthermore, proportionate differential increases in local government and healthcare funding in more deprived areas from 2001 to 2011 as part of a national programme of action to address inequalities were associated with improvements in infant mortality^13^ and reductions in inequalities in amenable mortality.^14^

However, there is limited evidence about whether, and if so, how far reductions in local public expenditure are associated with changes in emergency admissions. There is one study in the USA showing that increases in local government spending between 2007 and 2010 are associated with decreases in emergency hospitalisations between 2011 and 2014.^15^ Another study in England found that reductions in social care spending in older people were not associated with changes in average levels of avoidable and all-cause emergency admissions.^16^ However, this study neither accounted for expected increases in local public expenditure due to growing population needs nor did it examine impacts on socioeconomic inequalities in emergency admissions. In this study, we investigate whether local authority expenditure reductions were associated with inequalities between local authorities and neighbourhoods in avoidable and all-cause emergency hospitalisation considering the level of socioeconomic deprivation.

## Methods

We conducted an observational longitudinal analysis of socioeconomic inequalities in all-cause and avoidable emergency admissions between 2010 and 2017 and the association with local government funding changes. We analysed changes in the average level and the Absolute Gradient Index (AGI) of all-cause and avoidable emergency admissions at lower-tier local authority district (LAD) level. No ethical approval or patient consent procedures were required; this study involved analysis of aggregated area-based data. This study follows the STROBE (STrengthening the Reporting of OBservational studies in Epidemiology) reporting guidelines for cohort studies.^17^

### Setting

In 2017, the English local government had 152 upper-tier and 326 lower-tier local authorities. The upper-tier comprised 56 unitary authorities, 36 metropolitan districts in large cities, 33 London boroughs and 27 shire counties. The lower-tier comprised all unitary authorities, London boroughs and metropolitan districts plus 201 shire districts that are part of the shire counties.^18^

All funding for local government services is allocated at the lower-tier level, except for transport, highways, public health, and children and adult social care that are allocated at the upper-tier level.^18^

Each local authority is formed of small-area geographical units called lower-super output areas (LSOAs), which comprise between 400 and 1200 households and have between 1000 and 3000 residents.^19^ In England, there are 32 844 LSOAs, which can also be referred to as neighbourhoods.

### Data sources

#### Expenditure data

Local government expenditure at local authority district level for years 2007–2017 was extracted from the revenue outturn service expenditure summaries available from the Ministry of Housing, Communities and Local Government. Data from 2007 to 2009 were used to estimate expected expenditure growth (see below) and to identify the point at which expenditure reductions occurred.

Three measures of expenditure were used: total expenditure, services expenditure and social care expenditure. Total expenditure refers to the sum of all services except police and fire services, which have a different commissioning structure (details on expenditure categories in online supplemental figure S1). Services expenditure excludes education and public health services because responsibility for their commissioning changed during the observation period. Social care expenditure, usually referred to as long-term care in international literature, includes both adult and children’s social care.

10.1136/emermed-2022-212845.supp1 Supplementary data

For county districts, upper-tier expenditure was apportioned to the population of each constituent lower-tier authority and any expenditure at the lower-tier level for that item was added to the final value. For example, Cumbria is a shire county formed by six shire districts with a population of 498 375 in 2017. The expenditure allocated at the upper-tier level (ie, transport, highways, social care and public health) was divided by the population of the county and apportion to the population of each constituent district. All expenditures are expressed as expenditure per head. We adjusted expenditure data using Consumer Price Inflation annual average with 2016 as the reference year.

There were large across-the-board reductions in central government funding for local government in 2010. In these circumstances, it could be argued that the use of unadjusted expenditure figures could potentially misrepresent both the direction and the magnitude of change in expenditure relative to the needed expenditure after 2010. For example, if needed expenditure is growing year-on-year, then no change in unadjusted expenditure between 2009 and 2010 would represent a ‘reduction’ relative to the needed expenditure. Therefore, a reduction in real terms represents a larger loss of funding.

To allow for expected growth in need due to local population growth and ageing, we calculated the crude expenditure reduction or increase relative to predicted expenditure. We modelled predicted expenditure using a multilevel linear model with random intercepts and slopes with data for 2007 to 2009, where the outcome variable was expenditure (total, services or social care). This model had two levels, where the measurements over time belonged at level 1, and LADs were at level 2. Assuming that expenditure in 2009 met the local population’s need, we calculated the change of predicted versus current expenditure in 2010 in pounds per capita (more details in the online supplemental file 1).

#### Emergency admissions data

All-cause emergency admissions were defined as the total number of people admitted to hospital through an A&E department or referred for emergency admission directly by a general practitioner. Avoidable emergency admissions were defined as the indicator 106a of the Clinical Commissioning Group (CCG) Improvement and Assessment Framework (NHS England, 2018).^20^ Both rates are presented as indirectly standardised rates for age and sex per 100 000 inhabitants. The number of admissions per neighbourhood (ie, LSOA) was obtained from the Secondary Uses Service (SUS) dataset by year of discharge from NHSE/I. Total population per LSOA were obtained from the Office for National Statistics (ONS) mid-year population estimates.

#### Socioeconomic deprivation data

The Index of Multiple Deprivation (IMD) is a neighbourhood level (ie, LSOAs) relative measure of socioeconomic status that ranks each neighbourhood in England from 1 (most deprived) to 32 844 (least deprived) based on their score in seven domains: income; employment; education, skills and training; health and disability; crime; barriers to housing and services; and living environment.^21^

We used the rank of the IMD 2015 as a time-fixed measure of deprivation at neighbourhood level because previous analyses have shown that changes in neighbourhood deprivation are not associated with changes in emergency admissions.^22^ The IMD rank was transformed into a fractional rank between 0 (least deprived) and 1 (most deprived). LADs where deprivation spanned less than 60% of the 2015 IMD scale were excluded because, in these cases, measures such as the AGI, become unreliable. Deprivation at LAD level was calculated as the weighted mean of the IMD score for each LSOA in a LAD. Then, the scores were ranked to identify LAD’s quintile group of IMD deprivation. The highest ranked 20% by IMD is considered the most deprived.

### Analysis

We estimated the association between mean avoidable and all-cause emergency admissions (ie, between-area inequalities) and need-adjusted expenditure reductions between 2010 and 2017. To examine inequalities between neighbourhoods within a given local authority (ie, within-area inequalities by level of deprivation), we constructed equity indicators from 2010 to 2017 at the LAD level using the same methods as NHS England, except using ONS population data.^9^

The equity indicator for inequalities between neighbourhoods used by NHS England is the AGI. This is calculated using an ordinary least square (OLS) regression model of the relationship between neighbourhood-level deprivation and rates of avoidable and all-cause emergency admissions for each LAD for the years 2010–2017. The AGI represents the modelled gap (ie, the slope of the OLS regression) in emergency admissions between the most and least deprived neighbourhoods in England, if the local authority patterns were replicated nationwide. The mean (min-max) AGI for avoidable admissions in 2017 was 2288.7 (105.7–6221), while for all-cause emergency admissions, the AGI was 6909.3 (377.7–17 864.2).

We present descriptive data for 2010 and 2017, representing the first year where expenditure reductions were observed, and the last year included in the analysis. We chose the period between 2010 and 2017 for two reasons: (1) it was plausible that reductions in local government expenditure would have an effect on emergency admissions that could start appearing in 2010 and be detectable up to 2017; and (2) a 7-year period provided sufficient time to identify a trend in the data.

We used fixed-effects regressions to determine the association between the size of total expenditure reductions and between-area and within-area inequality trends in emergency admissions. The advantage of fixed-effects models is that they control for between-individuals time-invariant differences, so the coefficients cannot be biased due to omitted time-invariant characteristics.^23^ Because the distribution of deprivation within county districts is not homogeneous, we tested the robustness of our findings by running the analysis with and without these LADs. All analyses were conducted using Stata version/SE V.16.

### Patient and public involvement

It was not feasible to involve patients or the public in the design or conduct of this specific research. Members of the public are involved actively in the wider UNFAIR research programme and dissemination plans for all the research outputs (https://bit.ly/UNFAIRstudy).

## Results

We analysed data for 324 local authority districts, excluding Isle of Scilly (which only has one LSOA) and West Somerset (where deprivation spanned less than three deprivation quintile groups).

Local government expenditure increased steadily up to 2009, before falling from 2010 onwards. Reductions were smaller for social care (online supplemental figure S3). Between 2010 and 2017, total and services expenditure decreased, and social care expenditure increased (table 1). However, expenditure growth remained slower than predicted based on the rate of growth observed before 2010 for all types of expenditure, being more prominent for the most deprived LADs (table 1 and online supplemental figure S3). Services expenditure had greater reductions than social care for all levels of deprivation.

**Table 1:** Descriptive information for local government expenditure for years 2010 and 2017

	Local authority districts	
	**2010**	**2017**
Total expenditure per capita, £	1566 (1245–2733)	1284 (857–2217)
Services expenditure per capita, £	731 (520–1305)	678 (401–1418)
Social care expenditure per capita, £	385 (257–659)	426 (270–642)
Predicted total expenditure, £	1641 (1339–2782)	2110 (1615–3501)
Predicted services expenditure, £	782 (577–1505)	1012 (577–2593)
Predicted social care expenditure, £	405 (301–671)	548 (451–795)
Diff pred-current total expenditure (percent)	−4.6 (−19 to 7.2)	−39 (−64to −14)
Diff pred-current services expenditure (percent)	−6.3 (−33 to 13)	−30 (−70 to 17)
Diff pred-current social care expenditure (percent)	−5.1 (−23 to 44)	−22 (−54 to 4.8)
Diff pred-current total expenditure, £	−98.9 (−91.8 to −106)	−839.9 (−812.9 to −866.9)
Most deprived LADs, £	−120.1 (−99.2to −141)	−960.6 (−888.1 to −1033.2)
Least deprived LADs, £	−85.4 (−77.2 to −93.6)	−721.1 (−676.7 to −765.5)
Diff pred-current services expenditure, £	−67.4 (−62.3 to −72.6)	−368 (−346.6to −389.4)
Most deprived LADs, £	−83.3 (−67 to −99.6)	−504.8 (−447.4 to −562.2)
Least deprived LADs, £	−58.4 (−51.8 to −64.9)	−244.5 (−221.1 to −268)
Diff pred-current social care expenditure, £	−25.5 (−22.8 to −28.2)	−130.5 (−123.4 to −137.6)
Most deprived LADs, £	−27.7 (−18.8 to −36.6)	−151.3 (−128.6 to −173.9)
Least deprived LADs, £	−30.2 (−25.9 to −34.4)	−111.4 (−100.9 to −121.9)

All values reported as mean (min-max).

LADs, Local Authority Districts.

**Figure 1:**
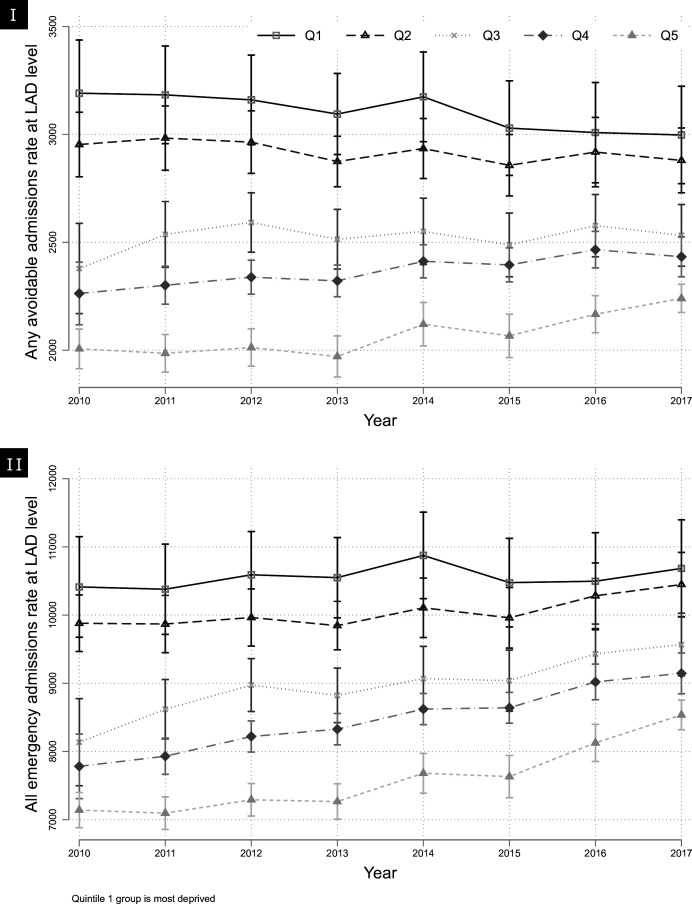
Trends in average (I) avoidable and (II) all-cause emergency admission rates by local authority deprivation quintile. LAD, local authority district.

During the period 2010–2017, all-cause and avoidable emergency admission rates were higher in more deprived LADs compared with less deprived ones (figure 1). All-cause and avoidable emergency admissions increased slightly for LADs in the least deprived quintile group. In contrast, there was a small decrease in avoidable admissions and an equivalent increase in emergency admissions for LADs in the most deprived quintile (table 2).

**Table 2:** Between-area and within-area inequalities in avoidable and all-cause emergency admissions between 2010 and 2017

	All-cause emergency admissions	Avoidable emergency admissions
Between-area inequalities, average rates per 100 000 population		
Most deprived LADs		
2010	10 413 (9675–11 151)	3191 (2945–3437)
2017	10 684 (9971–11 398)	2997 (2772–3223)
Least deprived LADs		
2010	7140 (6881–7399)	2006 (1915–2098)
2017	8536 (8319–8753)	2240 (2175–2306)
Within-area inequalities, absolute gradient index		
Most deprived LADs		
2010	8511 (7676–9345)	3179 (2864–3494)
2017	8421 (7735–9,108)	2904 (2642–3165)
Least deprived LADs		
2010	5290 (4890–5690)	1850 (1698–2003)
2017	6264 (5983–6990)	1980 (1817–2142)

All values reported as mean (95% CI).

LADs, Local Authority Districts.

Inequalities between neighbourhoods in avoidable admissions were higher in more deprived LADs between 2010 and 2017; however, these narrowed over the observation period. For all-cause emergency admissions, inequalities between neighbourhoods remained stable over the same period (see table 2 and online supplemental figure S2). Between-neighbourhood inequalities in avoidable admissions remained relatively stable for local authorities in the three least deprived quintile groups, while for all-cause emergency admissions, these inequalities increased (table 2).

**Figure 2:**
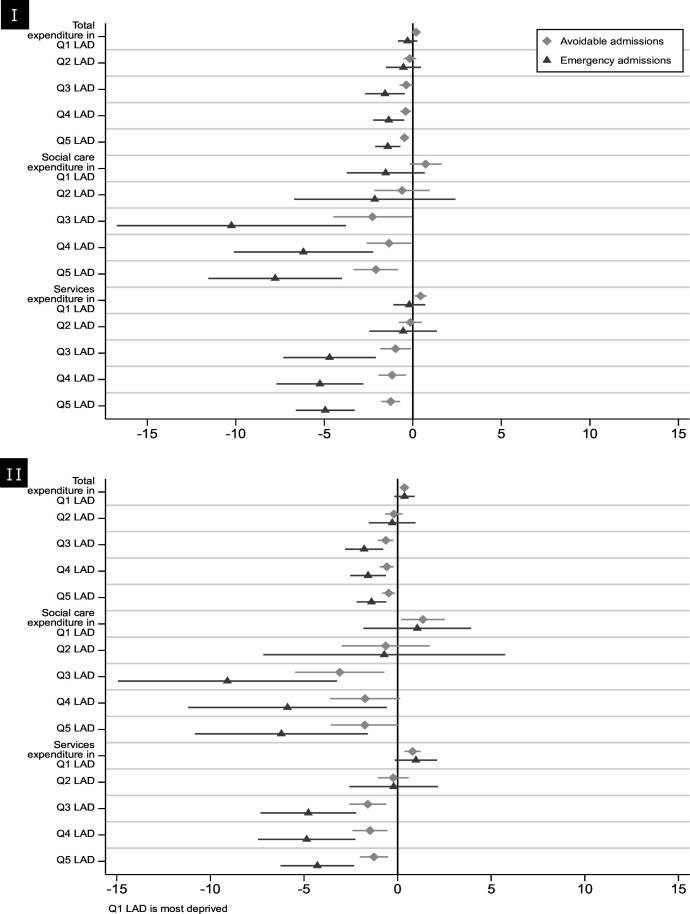
Impact of £1 increase in funding on (I) rates of avoidable and all-cause emergency admissions, and (II) Absolute Gradient Index for avoidable and all-cause emergency admissions, by local authority deprivation quintile. LAD, local authority district.

For local authorities in England’s three least deprived quintiles, reductions in local government expenditure (total, services and social care) were associated with increases in average rates of all-cause and avoidable emergency admissions. They were also associated with a widening of between neighbourhoods inequalities in all-cause and avoidable admissions (figure 2 and online supplemental table S1). This association remained significant for the least deprived LADs when we excluded county districts from the analysis (online supplemental table S2). There were no such associations for LADs in England’s two most deprived quintiles.

For a LAD in the least deprived quintile group, a £100 per head yearly reduction in total expenditure was associated with a yearly (95% CI) increase of 47 (22 to 73) avoidable admissions, 142 (70 to 213) all-cause emergency admissions and a yearly increase in the gap between the most and least deprived neighbourhoods of 48 (14 to 81) avoidable and 140 (60 to 220) all-cause emergency admissions.

## Discussion

After the 2008–2009 recession, the most deprived local authorities experienced the highest average rates of emergency admissions and greatest inequalities between neighbourhoods. Year-on-year spending reductions were associated with increases in average rates of all-cause and avoidable emergency admissions, and inequalities between neighbourhoods, but only in less deprived local authorities. In the most deprived local authorities, high average admission rates and wide inequalities persisted.

This study has not investigated causal mechanisms. However, local public expenditure reductions may increase emergency healthcare utilisation and healthcare inequality through multiple pathways centred on the social determinants of health. For example, insufficient social care provision places pressure on healthcare services, increasing emergency hospital use.^24^ Reduced spending on social support services might result in increased social isolation and loneliness, which are linked to higher emergency hospital admissions for stroke and cardiovascular disease.^25^ Reductions in housing services have been associated with a rise in homelessness,^26^ another known risk factor for emergency hospitalisation.^27^ Reductions in local government service expenditure may adversely impact services important to maintaining public health, including environmental health (important for water and food safety and infectious-disease control) and housing standards,^28^ which may result in increased emergency healthcare utilisation for gastrointestinal infections, respiratory, and cardiovascular diseases.^29^

There are several possible explanations for why year-on-year spending reductions were associated with increases in average rates of all-cause and avoidable emergency admissions, and inequalities between neighbourhoods, but only in less deprived local authorities.

First, supply constraints may have led to more stringent admission thresholds due to increasing demand for healthcare. When there is a limited number of beds and workforce capacity available in hospitals, physicians in the ED have to tighten admission criteria in the face of increasing demand to match activity to supply. It is plausible that any constraints would be felt most acutely in areas that experience high emergency admissions rates routinely, and our analysis demonstrates that local authorities in the most deprived quintiles experienced higher rates of emergency admissions over the observation period, and therefore, are subject to a ceiling effect, while the least deprived LADs have still spare capacity to admit more patients. Wyatt *et al* 30 analysed more than 20 million attendances to A&E between 2010 and 2015, finding that the case-mix adjusted probability of admission for walk-in adults fell by 22.9% during the study period. Moreover, they estimate that should the admission thresholds not have changed, admission would have been 11.9% higher in 2015. In an extension of this analysis, Wyatt e*t al* 31 found that the number of attendances with the highest odds of admission grew the fastest between 2010 and 2016, which led to an increase in the average acuity of patients attending A&E. Another potential explanation is that deprived LADs are accustomed to dealing with high levels of unmet population need; therefore, when faced with year-on-year financial reductions, these local authorities may have been more successful in mitigating the negative health consequences of these reductions falling on their most deprived neighbourhoods, thereby preventing any rise in average emergency admission rates and widening inequalities between neighbourhoods, compared with less deprived LADs. The fact that all-cause and avoidable emergency admissions increased more in the most deprived neighbourhoods within less deprived LADs (online supplemental figure S4 and S5) partially supports this hypothesis. Future research could explore to what extent this is true.

There is also the potential for substitution effects, which are difficult to measure. It is plausible that those facing more deprived circumstances will substitute their limited access to social care for informal care, while those in less deprived circumstances could pay privately for those services. The limited data available on the proportion of the population that pays privately or receives informal care prevented us from including these factors in our models.

Changes in avoidable emergency admission inequality in England are not explained by changes in neighbourhoods’ socioeconomic status over time (ie, gentrification) or changes in primary care supply or quality.^22^ There is currently a lack of understanding of potential explanations for changes in avoidable emergency admission inequalities,^3^ and our research goes some way to address this.

Local health and care systems cannot address healthcare inequalities in isolation. A deeper understanding of this landscape will help identify and measure more clearly the impact of action on the wider social determinants of health, and the role central government funding and policy decisions play in changing healthcare inequalities between local areas. This, in turn, will help local policymakers develop a more coordinated approach to improving health and well-being, and reducing health and care inequalities in their communities.

### Strengths and limitations

A strength of our analysis is that it uses the same deprivation-related inequality metric used by the NHS in England since 2016 to monitor local healthcare equity performance routinely, and as such, is of direct relevance to policy and practice partners. Our analysis extends previous analysis of this indicator^22^ as it is the first to analyse a contemporary timescale spanning the austerity period and to examine the association with local public expenditure changes.

The change in local government funding in England that occurred in 2010 created a natural policy experiment that allows estimation of the impact of this policy change on health outcomes. Fixed-effects panel regression enabled us to control for unobserved time-invariant confounders and known differences in economic trends. Our analysis using neighbourhood level data and aggregated at local authority level is more informative than an analysis of the national trend, while at the same time allows controlling for time-varying confounders that have a similar effect in all local authorities.

There were a number of limitations to this research. Our analysis did not consider the interaction between patient, hospital-level and local authority characteristics, or employ causal inference modelling to unpick complex causal pathways. For example, the analysis did not consider the influence of reductions in NHS spending, which had an increase of 1.3% per annum from 2010 to 2016, against increases in demand of over 3%.^32^ Since 2013, the proportion of NHS funding received by the most deprived areas has fallen relative to less deprived areas.^33^

Only two outcome variables (all-cause and avoidable emergency admissions) were considered. It is plausible that expenditure on social care may have larger effects on length of hospital stay rather than admission rates per se, so future analyses could explore different measures of inequalities in healthcare use. Furthermore, future research could examine the relationship between emergency attendances and local government funding, which would not be influenced by supply constraints.

Austerity policies implemented in 2010 were associated with increases in the level of emergency admissions and between-neighbourhood inequalities in emergency admissions in less deprived local authorities, while for the most deprived local authorities, emergency admissions and between-neighbourhood inequalities in emergency admissions remained high and persistent. We cannot, however, conclude that year-on-year local public expenditure reductions only had a detrimental impact on healthcare inequalities in less deprived local authority areas. Disentangling the effects of individual, local government and health services factors on inequalities in avoidable hospital admission is complex. More granular data allowing for the interaction between patient, hospital-level and local authority characteristics, and using more sophisticated causal inference modelling to unpick the complex causal pathways are required.

## Data Availability

Data may be obtained from a third party and are not publicly available. This study used data aggregated at lower super output area level from the Secondary Uses Service dataset. We cannot publish this dataset, but other researchers can use it by applying for access to NHS England. All other datasets used are publicly available.
