# Supplementary material for: Are local public expenditure reductions associated with increases in inequality in emergency hospitalisation? Time-series analysis of English local authorities from 2010 to 2017
Source: Emerg Med J. 2024 Jun 13;41(7):389–96. doi: 10.1136/emermed-2022-212845 (PMC11228196; doi:10.1136/emermed-2022-212845)

Supplementary file to "Are local public expenditure reductions associated with increases in inequality in emergency hospitalisation? Time-series analysis of English local authorities from 2012 to 2017"

Contents

1 Primer on the AGI of inequality	1
2 Statistical appendix	2
3 Tables	
Table S1	3
Table S2	4
4 Figures	
Figure S1	5
Figure S2	6
Figure S3	7
Figure S4	8
Figure S5	9
Figure S6	10

Primer on the AGI of inequality

For full technical details see this journal article (https://doi.org/10.1016/j.socscimed.2018.01.004).

What is an AGI?
An AGI is an **Absolute Gradient Index** of inequality.
It is the modelled gap in outcomes between the most and least deprived neighbourhoods in the country. You can use any outcome that can be broken down by deprivation groups, for example, avoidable emergency admissions, GPs per population, rates of early stage cancer diagnosis, waiting times for psychological therapies, and many others.

Why a "modelled" gap rather than an "actual" gap?
Because it summarises information about everyone in the whole population, not just a few people at the two extremes. Actual gaps between extreme groups can be unstable and misleading.

Why the most and least deprived "in the country" rather than "in my local area"?
Because local inequality in your area can then be compared with local inequality in other areas on a like-for-like basis. For instance, the most deprived fifth of people in Buckinghamshire are considerably better off than the most deprived fifth in Liverpool. Inequality based on national deprivation rank can be compared between local areas, but not inequality based on local deprivation rank.

How is the modelling done?
Simple linear regression at neighbourhood level. We take all the neighbourhoods (technically "Lower Layer Super Output" areas) within your locality (e.g. clinical commissioning group or local authority). We then graph an outcome versus the national deprivation rank for each neighbourhood, and draw a straight line through those points considering the local population size (bigger neighbourhood have more influence on the direction of the line). That straight line is the "social gradient" in outcomes. The "modelled gap" is the difference between the top and bottom of the social gradient, representing the most and least deprived neighbourhoods in the country.

What about simpler equity measures?
Useful simple equity measures for non-specialists include:

- The actual gap between best- and worst-off fifth (or tenth) based on national deprivation
- The trend in outcomes for the worst-off fifth (or tenth) based on national deprivation

When is the AGI misleading?
The AGI is unreliable for localities with a narrow deprivation range (fewer than 3 quintile groups) such as Bradford City CCG, where almost everyone is in the most deprived national quintile group. In such cases, it is more helpful to look at trends in the worst-off quintile group(s).

Statistical Appendix

- Equation multilevel linear model for predicting expected expenditure

$$= \beta_{0j} + \beta_{1j}year_{ij} + U_{0j} + U_{1j}\, time_{ij} + \varepsilon_{ij}$$

- Command for estimating the multilevel linear model for predicting expected expenditure

mixed *expenditure-per-capita year* || *LAD_id*: *year*, mle covariance(unstructure)

- Example:

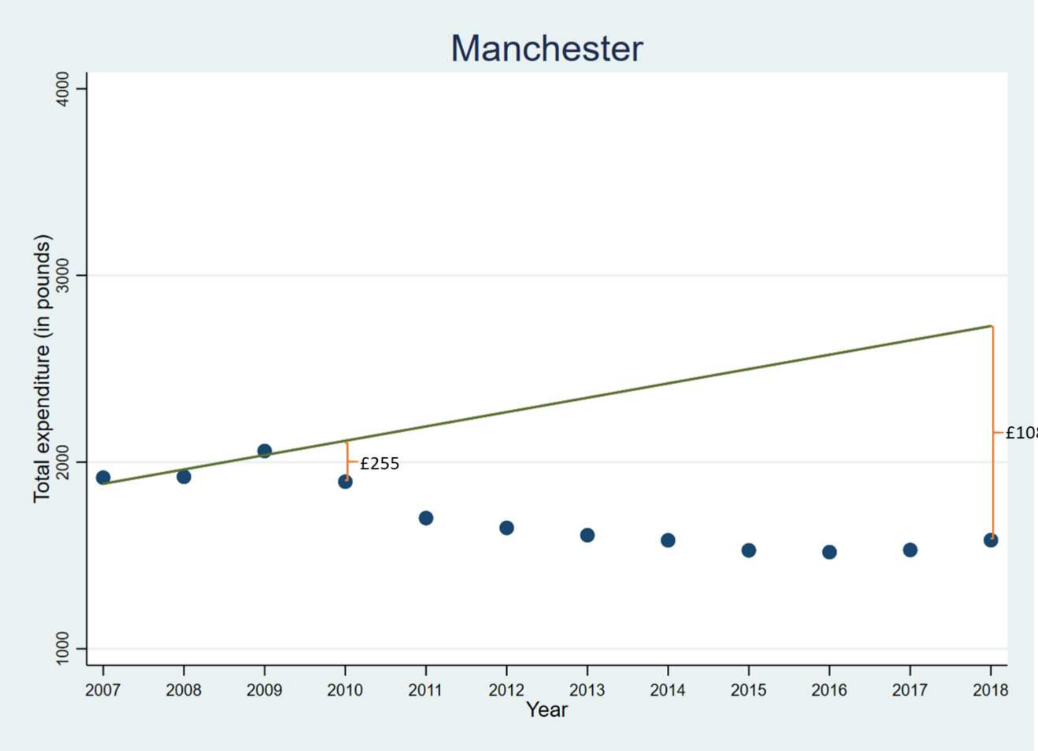

- Command for estimating the fixed effects panel linear regression:

xtset LAD_id year
xtreg *emergency-admissions need-adjusted-expenditure-change##IMD15_quintile_group*, fe vce(robust)

- Test of correlation between outcome variable and error term

	Total expenditure	Services expenditure	Social care expenditure
Rate of avoidable admissions	-0.56	-0.56	-0.45
Rate of emergency admissions	-0.41	-0.49	-0.37
AGI of avoidable admissions	-0.52	-0.55	-0.39
AGI of emergency admissions	-0.50	-0.53	-0.42

Castro-Ávila AC, *et al. Emerg Med J* 2024; 41:389–396. doi: 10.1136/emermed-2022-212845

Table S1. Coefficients of the fixed-effects linear regression models

	Avoidable emergency admissions Coefficient (95% CI)		All-cause emergency admissions Coefficient (95% CI)	
	**Rates**	**AGIs**	**Rates**	**AGIs**
**Total expenditure**				
**Most deprived**	0.19 (-0.03 - 0.40)	0.36 (0.12 - 0.61)	-0.30 (-0.84 - 0.24)	0.37 (-0.18 - 0.91)
**Quintile 2**	-0.17 (-0.53 - 0.19)	-0.20 (-0.66 - 0.27)	-0.53 (-1.53 - 0.46)	-0.29 (-1.54 - 0.96)
**Quintile 3**	-0.37 (-0.75 - 0.01)	-0.63 (-1.05 - -0.21)	-1.57 (-2.69 - -0.45)	-1.79 (-2.81 - -0.77)
**Quintile 4**	-0.40 (-0.70 - -0.10)	-0.58 (-0.95 - -0.21)	-1.36 (-2.24 - -0.48)	-1.58 (-2.53 - -0.62)
**Least deprived**	-0.47 (-0.73 - -0.22)	-0.48 (-0.81 - -0.14)	-1.42 (-2.13 - -0.70)	-1.40 (-2.19 - -0.60)
**Services expenditure**				
**Most deprived**	0.43 (0.09 - 0.77)	0.80 (0.37 - 1.23)	-0.20 (-1.10 - 0.70)	0.98 (-0.16 - 2.11)
**Quintile 2**	-0.14 (-0.79 - 0.51)	-0.23 (-1.05 - 0.59)	-0.55 (-2.46 - 1.36)	-0.21 (-2.58 - 2.16)
**Quintile 3**	-0.97 (-1.84 - -0.10)	-1.59 (-2.58 - -0.60)	-4.70 (-7.32 - -2.09)	-4.77 (-7.33 - -2.21)
**Quintile 4**	-1.17 (-1.95 - -0.40)	-1.47 (-2.41 - -0.53)	-5.25 (-7.70 - -2.79)	-4.86 (-7.46 - -2.25)
**Least deprived**	-1.25 (-1.78 - -0.72)	-1.26 (-2.02 - -0.50)	-4.95 (-6.61 - -3.28)	-4.29 (-6.25 - -2.32)
**Social care expenditure**				
**Most deprived**	0.72 (-0.18 - 1.63)	1.35 (0.19 - 2.51)	-1.53 (-3.74 - 0.67)	1.05 (-1.83 - 3.93)
**Quintile 2**	-0.61 (-2.17 - 0.95)	-0.64 (-2.99 - 1.72)	-2.15 (-6.71 - 2.41)	-0.72 (-7.17 - 5.74)
**Quintile 3**	-2.28 (-4.50 - -0.06)	-3.09 (-5.47 - -0.7)1	-10.25 (-16.72 - -3.77)	-9.09 (-14.95 - -3.24)
**Quintile 4**	-1.35 (-2.61 - -0.09)	-1.74 (-3.61 - 0.14)	-6.18 (-10.12 - -2.24)	-5.88 (-11.19 - -0.58)
**Least deprived**	-2.09 (-3.35 - -0.82)	-1.75 (-3.57 - 0.07)	-7.77 (-11.56 - -3.99)	-6.21 (-10.83 - -1.60)

Table S2. Coefficients of the fixed-effects linear regression models excluding county districts

	Avoidable emergency admissions Coefficient (95% CI)		All-cause emergency admissions Coefficient (95% CI)	
	**Rates**	**AGIs**	**Rates**	**AGIs**
**Total expenditure**				
**Most deprived**	0.18 (-0.05 - 0.41)	0.38 (0.12 - 0.65)	-0.27 (-0.87 - 0.32)	0.48 (-0.09 - 1.05)
**Quintile 2**	-0.04 (-0.50 - 0.41)	-0.05 (-0.65 - 0.55)	-0.19 (-1.45 - 1.07)	0.15 (-1.48 - 1.78)
**Quintile 3**	-0.46 (-1.12 - 0.19)	-0.62 (-1.32 - 0.08)	-1.78 (-3.79 - 0.24)	-1.72 (-3.46 - 0.01)
**Quintile 4**	-0.51 (-1.13 - 0.12)	-0.68 (-1.45 - 0.09)	-1.86 (-3.92 - 0.19)	-2.03 (-4.25 - 0.18)
**Least deprived**	-0.68 (-1.16 - -0.20)	-1.08 (-1.82 - -0.94)	-1.94 (-3.66 - -0.23)	-2.59 (-4.37 - -0.81)
**Services expenditure**				
**Most deprived**	0.42 (0.06 - 0.78)	0.81 (0.36 - 1.26)	-0.14 (-1. 09 - 0.81)	1.10 (-0.07 - 2.28)
**Quintile 2**	0.09 (-0.64 - 0.82)	0.02 (-0.93 - 0.96)	0.14 (-1.93 - 2.21)	0.52 (-2.19 - 3.22)
**Quintile 3**	-1.24 (-2.64 - 0.18)	-1.63 (-3.22 - -0.04)	-5.20 (-9.55 - -0.86)	-4.70 (-8.82 - -0.56)
**Quintile 4**	-2.12 (-4.34 - 0.10)	-2.28 (-4.80 - 0.24)	-9.18 (-16.54 - -1.82)	-7.53 (-14.98 - -0.07)
**Least deprived**	-1.72 (-2.92 - -0.52)	-2.41 (-4.17 - -0.66)	-6.38 (-11.13 - -1.63)	-6.24 (-10.87 - -1.61)
**Social care expenditure**				
**Most deprived**	0.68 (-0.28 - 1.63)	1.39 (0.17 - 2.61)	-1.39 (-3.72 - 0.94)	1.46 (-1.50 - 4.41)
**Quintile 2**	-0.06 (-1.85 - 1.73)	0.03 (-2.76 - 2.82)	-0.40 (-5.68 - 4.87)	1.42 (-6.24 - 9.08)
**Quintile 3**	-3.26 (-7.02 - 0.50)	-3.27 (-7.26 - 0.72)1	-12.43 (-23.84 - -1.02)	-9.32 (-19.03 - 0.40)
**Quintile 4**	-1.41 (-3.91 - 1.09)	-2.05 (-6.06 - 1.95)	-6.61 (-16.18 - 2.95)	-6.02 (-18.47 - 6.42)
**Least deprived**	-3.55 (-6.45 - -0.65)	-4.76 (-9.55 - 0.04)	-12.63 (-24.31 - -0.96)	-12.83 (-23.56 - -2.10)

Castro-Ávila AC, *et al. Emerg Med J* 2024; 41:389–396. doi: 10.1136/emermed-2022-212845

Figure S1 Local Authority Service Expenditure by Category, 2017-18

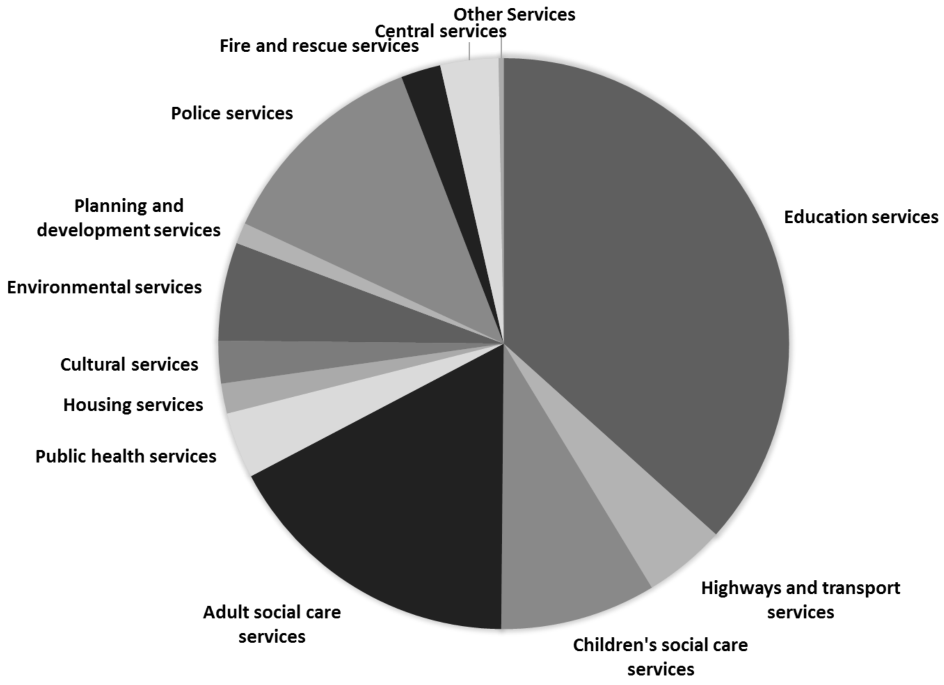

Source: Ministry of Housing Communities and Local Government (2019).
Note: This excludes capital expenditure and housing benefit, which is not a local government service but a cash benefit administered by local government under central government rules.

Figure S2 - Trends in Absolute Gradient Index of Inequality (AGI) between neighbourhoods within a local authority for (I) avoidable and (II) all-cause emergency admissions, by local authority deprivation quintile.

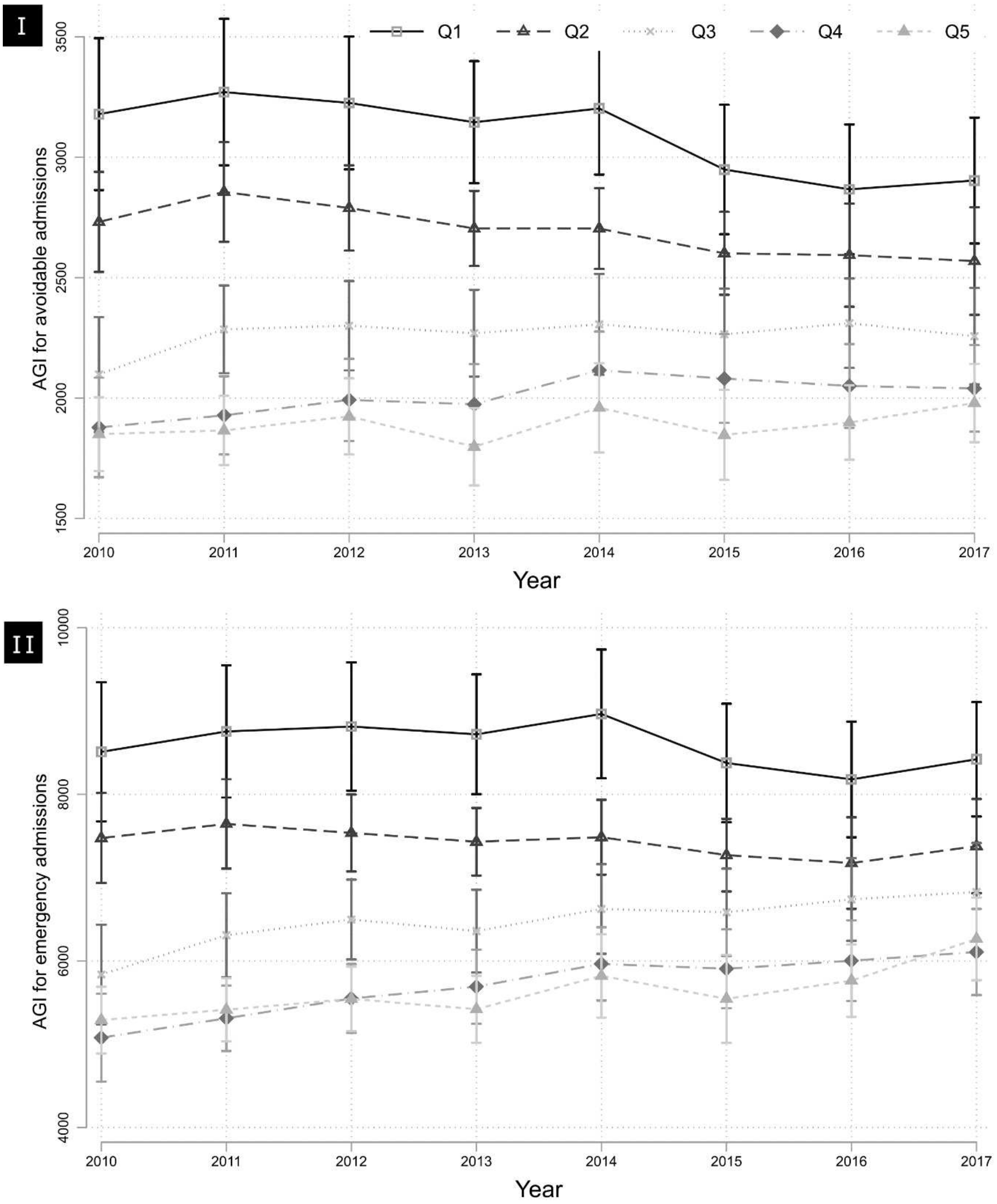

Quintile 1 group is most deprived

Figure S3- Trends in local government expenditure per head for (I) total, (II) services, and (III) social care, by local authority deprivation quintile.

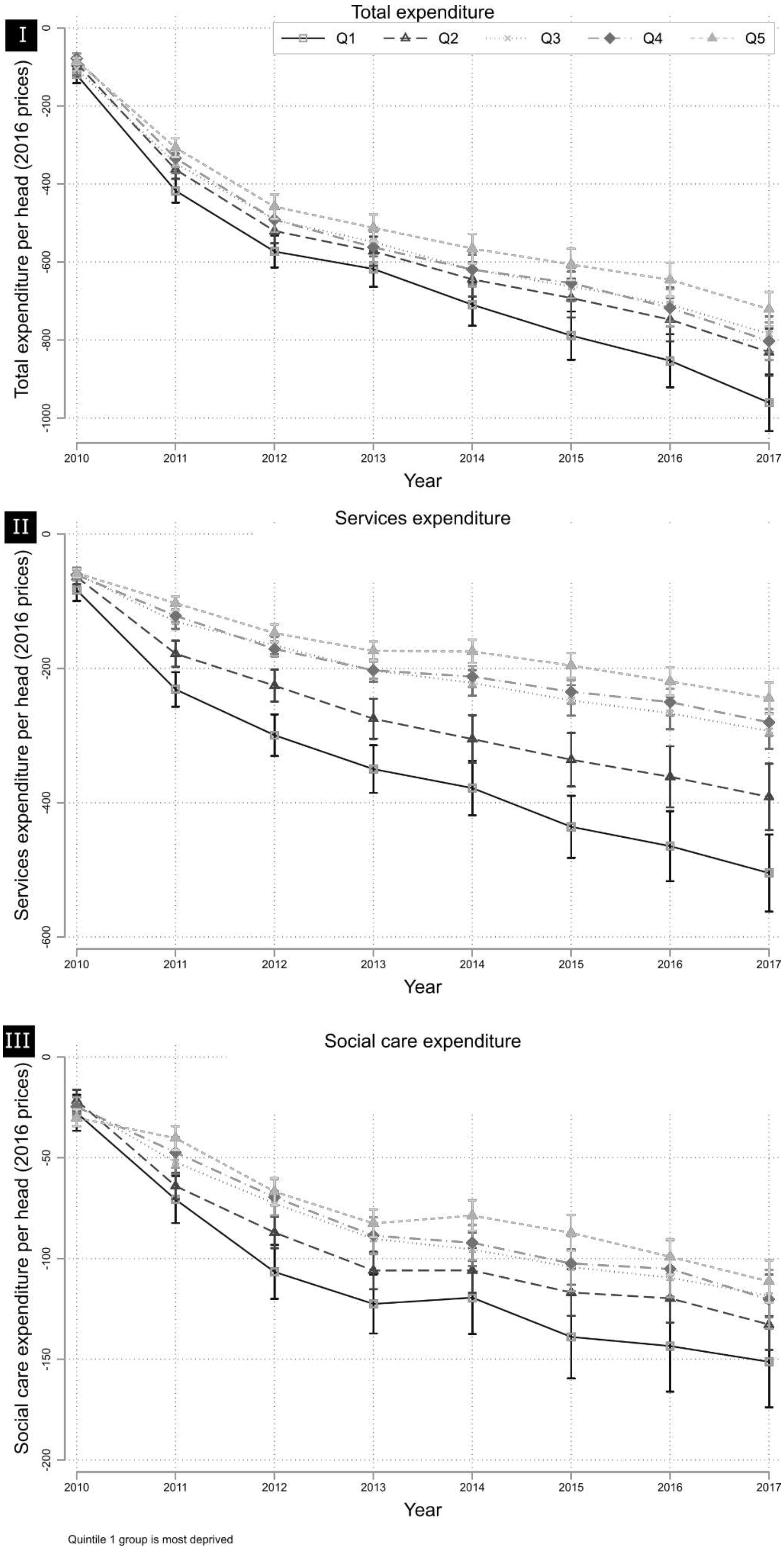

Figure S4- Comparison of avoidable emergency admission rates for years 2010 and 2017 by quintile groups of deprivation of lower super output areas (LSOA) for (I) most deprived LADs, and (II) least deprived LADs.

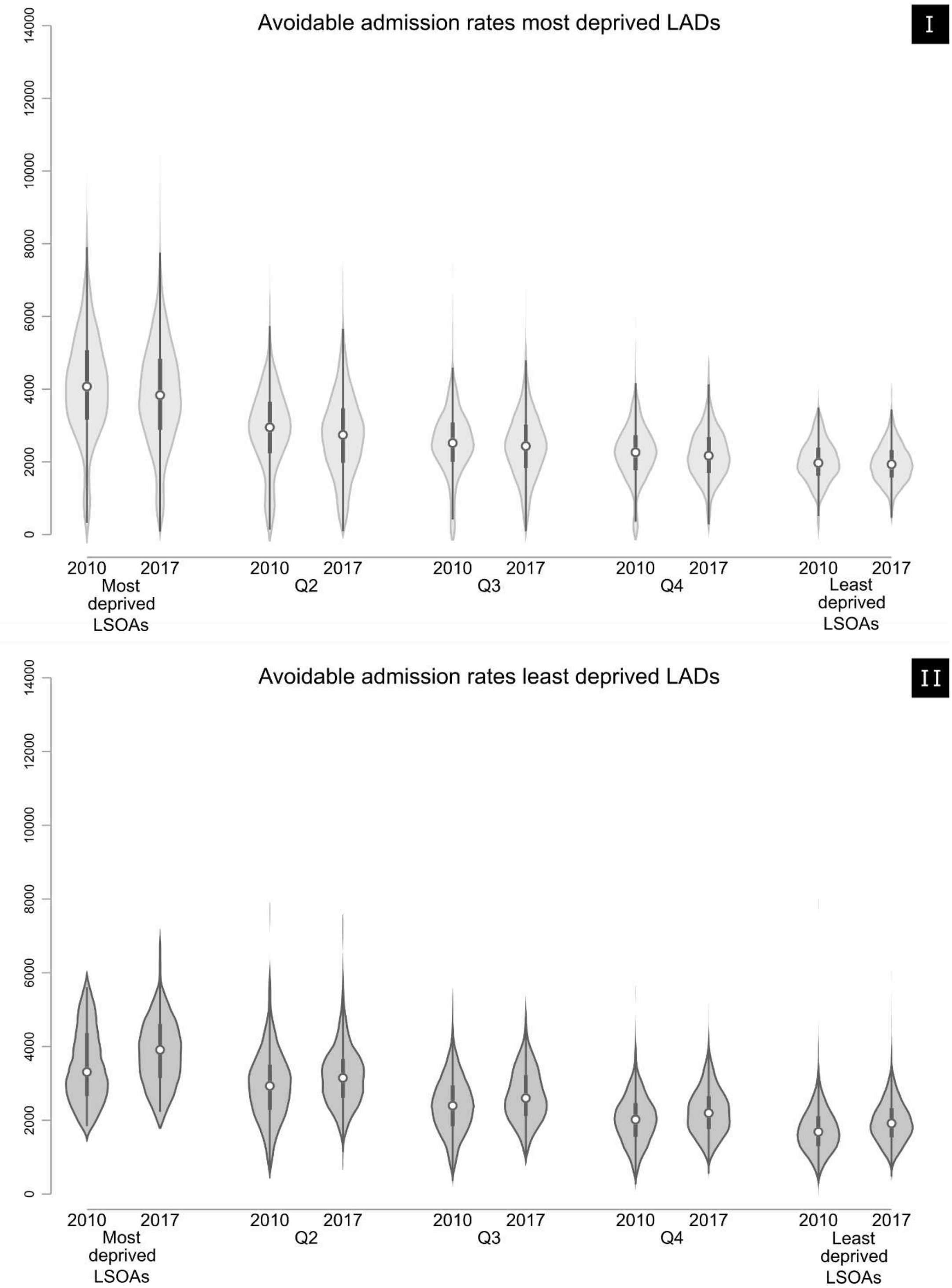

Indirectly age-sex standardised rates per 100,00 population

Castro-Ávila AC, *et al. Emerg Med J* 2024; 41:389–396. doi: 10.1136/emermed-2022-212845

Figure S5- Comparison of all-cause emergency admission rates for years 2010 and 2017 by quintile groups of deprivation of lower super output areas (LSOA) for (I) most deprived LADs, and (II) least deprived LADs.

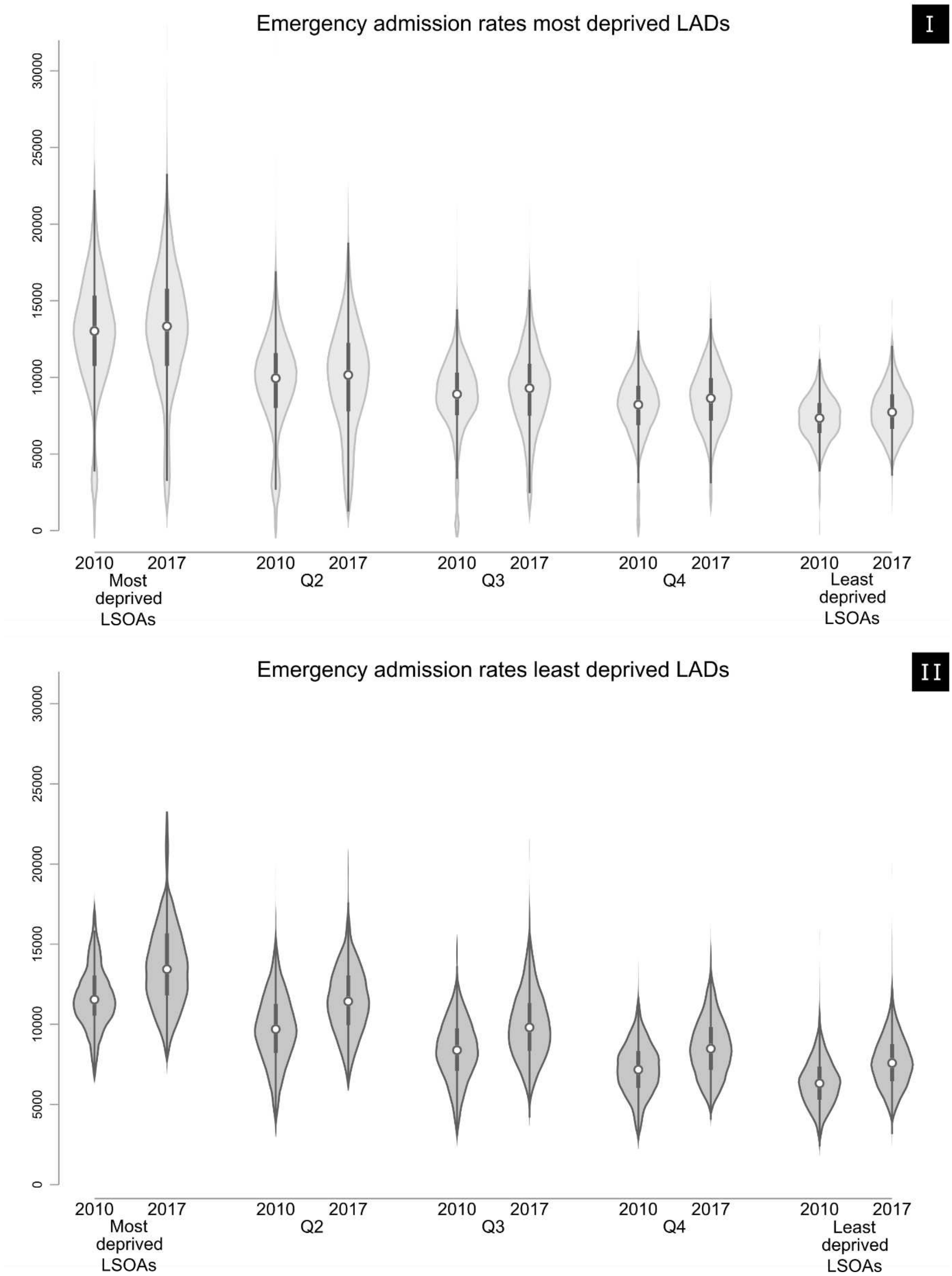

Indirectly age-sex standardised rates per 100,00 population

Figure S6- Maps for 2017 for (I) LADs deprivation, (II) Total expenditure reductions, (III) Standardised rates of any avoidable admissions per 100,000 population, and (IV) Absolute gradient index of inequality for any avoidable admissions.

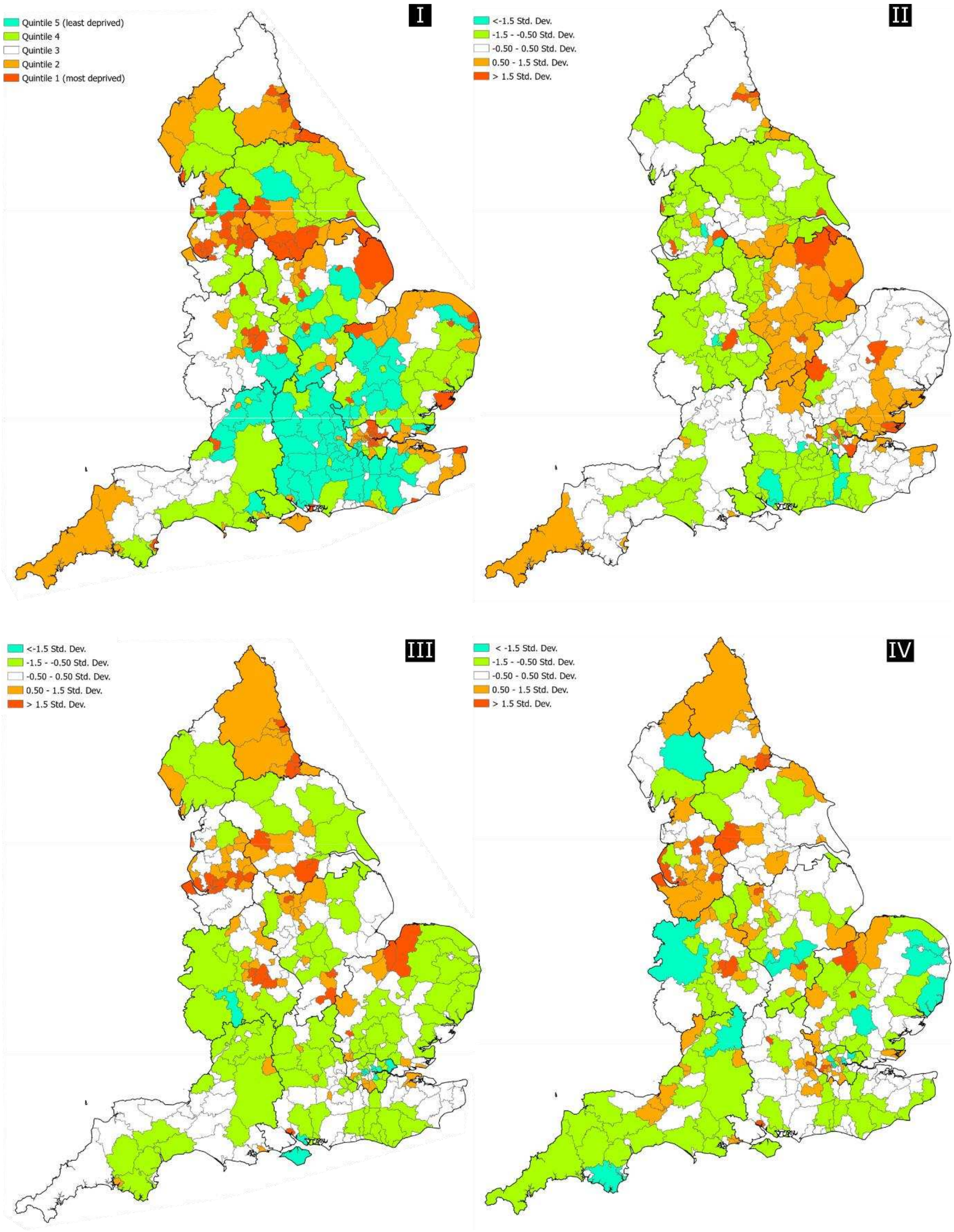

Supplement: Supplementary data [file emermed-2022-212845supp001.pdf]
